# *GLocal*: A global development dataset of subnational administrative areas

**DOI:** 10.1038/s41597-024-03539-y

**Published:** 2024-08-08

**Authors:** Jose Morales-Arilla, Shreyas Gadgin Matha

**Affiliations:** 1 Research Professor, Tecnologico de Monterrey, Escuela de Gobierno y Transformación Pública, Ciudad de México, 03700 Mexico; 2 Senior Computational Social Scientist, The Growth Lab at Harvard University, Cambridge, MA 02138 USA

**Keywords:** Government, Economics

## Abstract

The purpose of the *GLocal* dataset is to enable research in international development that requires both global scope and local precision. Leveraging modern geospatial analysis tools, we process a diverse array of sources to provide researchers with a growing set of economic, demographic, ecological and socio-political variables for geographic units relevant to public policy. We provide separate data files for different levels of administrative and periodic aggregation, along with ad-hoc files with more detailed information on specific topics. In this data descriptor paper, we discuss both our data processing methodologies and validation pipelines, and provide a short case study to illustrate the research potential of the dataset. We also introduce a simple web app, glocal.streamlit.app, which offers a user-friendly interface for exploring and visualizing the dataset. Given the growing number of public and granular sources of relevance for international development research, we hope to continue adding features and expand the *GLocal* dataset in the future.

## Background & Summary

Research on international development often requires subnational comparisons across different countries. One such example is the development of comparative studies of regions that are unique within their own countries, but that share noticeable similarities with other regions outside their national borders. For example, in order to analyze patterns of development and extraction in the Colombian Amazon, the most adequate benchmarks may not be found in other Colombian regions, but in Amazon regions in other countries^1^. More broadly, research questions of global importance often depend on subnational variation to abstract from potential confounders. For instance, studies focusing on the developmental impacts of regional and ethnic favoritism often leverage local variation in relative affiliation to national leaders^2,3^. Finally, development researchers and policy analysts often find themselves lacking fundamental quantitative inputs in settings with underdeveloped statistical sources^4^. These problems compound as national statistical agencies do not coordinate the timing and methodologies with which they measure development outcomes of interest, forcing researchers to develop ad-hoc solutions that often rely on specialized geospatial analysis skills outside of the toolbox of most development researchers and analysts.

To address these constraints, we have developed a structured dataset that provides subnational economic, demographic, ecological and socio-political indicators for all countries in the World. This *"GLocal”* dataset leverages a diverse and growing array of granular sources, processing them with modern geospatial analysis tools to create aggregates at consistent geographic and temporal levels. Importantly, data are aggregated at different levels of subnational administrative boundaries, which are of special interest for the purpose of policy-making. The resulting datasets are immediately available for download and analysis. More specifically, we provide 14 files corresponding to the aggregations - 9 files for each combination of three administrative and three periodicity levels, 3 files with all variables annualized for convenience at each administrative level (monthly data aggregated to yearly, cross-section data assigned to the year of observation), and 2 ad-hoc files with detailed information on agro-ecological crop suitability and mineral deposits. We also provide some supporting data containing a codebook and some information related to the administrative areas, for convenience. Overall, the GLocal datasets provide internationally comparable and locally precise data on nighttime lights (VIIRS, DMSP and Harmonized)^5–9^, land coverage types^10,11^, population and population density^12^, protests and violent events^13^, temperature and rainfall^14–16^, terrain topology^17,18^, trade infrastructure^19,20^, deforestation^21^, agricultural output and suitability^22^, relationship to capital city and water bodies^23,24^, mineral deposits and gas flares^25,26^, road network density^27^, reach of telecommunications services^28,29^, clean energy potential^30^, carbon emissions and air quality^31,32^. While our hope is to further expand the set of comparable and precise features available through the GLocal dataset, we believe the current set should enable researchers working on some of the most consequential topics in economic development: Economic growth, urbanization, deforestation, infrastructure, conflict and climate change. Importantly, the final list of variables in the dataset was determined considering licensing restrictions and the international comparability of original data sources. For instance, licensing constraints prevented us from creating GLocal aggregations from the Armed Conflict Location and Event Data Project (ACLED)^33^. Moreover, comparability issues led us not to incorporate data aggregations of local statistical agencies, even if broadly available through systems like IPUMS International^34^.

The rest of this data descriptor continues as follows: First, we provide an exhaustive list of the inputs that we use for the creation of the dataset, and outline the specific geospatial aggregation routines performed for each type of dataset. Second, we provide the data records that can allow readers to download and use the data. Third, we outline the technical validation exercises that we performed to confirm the quality of the datasets for each specific source. Fourth, we provide usage notes for development researchers to make use of the data, and introduce short case studies to highlight the potential use of this source. Finally, we outline how we make our processing and validation code available to all users.

## Methods

### Administrative aggregation

In order to develop the GLocal dataset, we first needed to select a standard for the spatial reach of geographic units throughout the world. While similar efforts have focused on apolitical territorial “grids” that are exogenous to economic, political and cultural features of interest^35,36^, we focus on established administrative areas. We do so because these are of special interest for policy-making purposes, as they align with the separation of sovereignty and policy-making authority, and constitute a shared references for citizens and governments on the classification of different territories. We used the Database of Global Administrative Areas (GADM) in its 3.6 version^37^. We took GADM polygon shapefiles for administrative units at levels 0 (e.g. Republics, Kingdoms), 1 (e.g. States, Provinces), and 2 (e.g. Municipalities, Districts), and calculated their respective geometric centroids. Moreover, we used population data from 2020^38^ to also calculate the respective population-weighted centroids.

With polygon borders and point centroids characterizing each administrative area, we have all the inputs necessary to link all locations to the different data sources. Table 1 provides a taxonomy to organize the information available in the GLocal dataset into distinct categories. The different categories are classified according to the original data type (A: Points, B: Lines/Polygons, or C: Rasters) and their topic (I: Ecological, II: Political/Demographic, or III: Economic). The accompanying data codebook provides specific details on all the variables created, including units, available time period, periodicity, licensing and terms of use, source URLs and citations. Importantly, the codebook is organized according the categories shown in Table 1. For example, variables on Nighttime lights are the first subtopic of variables of Type C and Topic III. Hence, these variables can be found in the codebook under category C.III.1.

**Table 1:** Taxonomy of information in the GLocal dataset.

Topic	I - Ecological	II - Political-Demographic	III - Economic
Data type			
A - Points		1. Capital cities 2. Distance to Capital 3. Protest and violence	1. Trade Infrastructure 2. Mineral deposits 3. Telecom antennas 4. Time to infrastructure 5. Time to markets
B - Lines/Polygons	1. Water bodies 2. Coastline	1. Land border	1. Road density 2. Gas flares
C - Rasters	1. Landcover 2. Rain and temperature 3. Terrain 4. Clean energy potential 5. Air quality	1. Population 2. Population density	1. Nighttime lights 2. Agricultural production 3. Deforestation 4. Telecom signal

### Point sources

Point sources identify the geolocation of different events or elements of interest. For these sources, we were interested in either counting the total number of points that intersect with each administrative polygon, creating a binary marker for the presence of such intersections, or calculating either the distances or the travel times between each administrative centroid and its nearest point. All these calculations were performed in R with standard functions from the sf package^39^ that intersect points to polygons or calculate geodesic distances between points using the same geographic projection. The notable exception was the calculation of driving times to trade infrastructure and markets. In this instance, we used a least-cost path algorithm on Google Earth Engine following the methodology and data outlined in^40^ to assess driving times between the pixels in each administrative area and different points of interest (e.g. Cities, Large Cities, Ports, Airports), and then obtained the median travel time to various resources from each administrative area.

### Line/Polygon sources

Similar to point-coded information, we were interested in the intersection of administrative polygons and the distance between administrative centroids with the lines and polygons characterizing different sources. The relevant geodesic distances were calculated using standard functions from the sf package^39^ in R. A key exception was the calculation of the road density in an administrative area. In this case, we took worldwide road network data from OpenStreetMaps via the GRIP global roads database^41^, and calculated the total length of roads that fall entirely within each administrative unit. For the purpose of computational efficiency at a global scale, we used the Mollweide equal-area projection in calculating the length of road networks within each administrative polygon.

### Raster sources

Raster-coded sources incorporated in the GLocal dataset are either categorical or continuous. Categorical rasters classify each pixel into types. In these cases, we calculated the total area in each administrative unit that falls in each of the relevant categories. Continuous rasters provide numeric information about the magnitude of a certain phenomena in each pixel. We aggregated continuous rasters into total sums or area-weighted means or medians for each administrative unit. The dataset’s codebook identifies the specific aggregation function performed for each of the raster aggregation variables. Rasters were ingested using the terra and raster packages in R^42,43^. All raster aggregations were performed with zonal statistics functions in R using the exactextractr package^44^. The values of border pixels were split according to the share of the pixel’s area that falls in each administrative polygon. That is, our methodology currently assumes that the data for a pixel is uniformly distributed across the pixel, which is a simplification. A notable exception is that of Custom VIIRS Nighttime Lights and the land cover variables. The custom cleaning and aggregation routines pursued in this case were performed on Google Earth Engine, which fully assigns border pixels’ values to the administrative polygons that coincide with each pixel’s centroid. For more coarse resolutions, since vector boundaries often intersect with pixels, these simplifications can introduce some inaccuracies. We have used the most detailed resolution available to reduce these inaccuracies, but this remains a limitation.

## Data Records

The GLocal datasets are available in the Harvard Dataverse: 10.7910/DVN/6TUCTE^45^. The repository includes 14 data files containing GLocal aggregations, and some supporting data. Of the 14, there are nine files that correspond with each combination of three levels of administrative detail (GID0, GID1 and GID2) and three periodicity levels (annual, monthly, and cross-section). Cross-section refers to sources that are either fixed characteristics (such as elevation) or were just measured once. For data measured only once, the year of measurement is specified in the data codebook. There are three annualized files with information that has yearly or multi-year periodicity, and monthly files with sources that provide monthly variation. The repository has two additional cross-section datasets with information about the agroecological suitability of different crops, and the presence of deposits of different minerals. We provide these files in both CSV and Parquet data formats. Additionally, the repository includes summary and validation pdf files that outline the characteristics of each variable in the dataset. Finally, the users will find a thorough codebook of variable characteristics.

We have included selected summary statistics (min, max, median, mean) by country of each variable at the GADM levels 1 and 2 as part of the output from the data validation process in order to allow the user to get a high-level understanding of the data. These summary statistics along with the code required to produce them are available in the GitHub repository.

## Technical Validation

Data validation was conducted in three stages for each of the three levels of geographic specificity - integrity, completeness and aggregation consistency.

Integrity examined that each region had exactly one row for each year, even if no data was present in the row. In short, it assessed if the data set was “rectangular." In the most recent iteration of the data, all regions in all temporal and spatial aggregations are present for all periods, indicating perfect integrity.

Completeness conducted two tests. The first was assessing the prevalence of NA’s for variable in a given region within the variables’ time range. For instance, the “Rain - GPCP" variable has data for every year in the 1979 - 2021 range, and therefore should have zero NA’s in this range.

The second test implemented simple internal validity measures to ensure the consistency of data - providing summary statistics like minima, maxima, means and medians for each variable in each data set. Every observation was compared against indicators for anomalies, such as a nightlight count below zero, which would be impossible, or elevations above or below geographic extremes, to ensure raster processing errors were rectified. The results for both tests for each variables are available in PDF form in the validation section of the code repository.

Finally was an aggregation consistency check - making sure that, for relevant statistics, the higher level geographic areas were reflective of the sum or geographically weighted average of their constituent parts. For instance, GID0 areas should have a nightlight count value equal to the sum of all of their GID1 subdivisions, and each GID1 subdivision should be the sum of its GID2 subdivisions.

Results indicated that for relevant variables, aggregation was within 1 percent of expected values, with over half being within .01 percent. Specifically, GDELT values do not aggregate uniformly across levels due to the fact that the geolocation assigned to some events in the original data are set to be representative of broader levels of geographic precision. Therefore, they were excluded from aggregation consistency checks. A table summary of the consistency of aggregation for each variable is available in the validation section of the code repository.

**Fig. 1:**
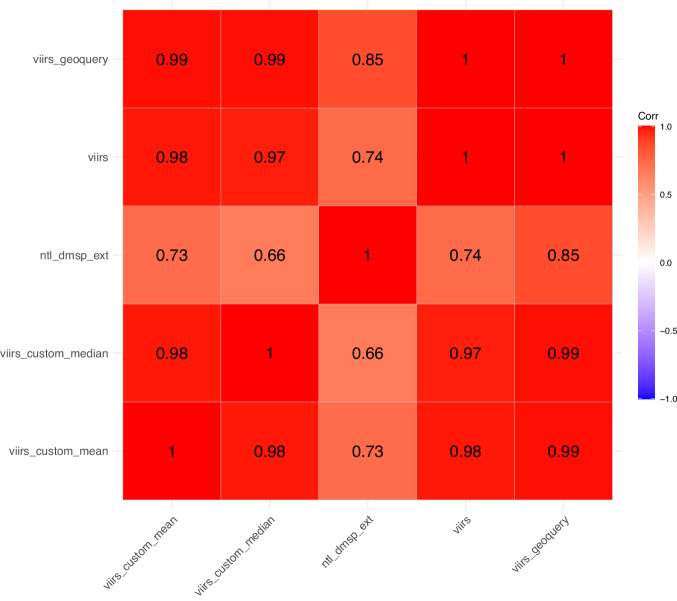
Correlation Plot of VIIRS Nightlight Measures from GLocal and Geoquery.

We also conducted an external validity check against nighttime lights data aggregated by GeoQuery^46^. GeoQuery is a research initiative hosted by the AidData lab at William & Mary, which also facilitates the aggregation and analysis of certain geospatial features for academic research. GeoQuery uses different administrative boundaries from GLocal, except for the USA, where we are able to compare the two at the state level (GID-1). Figure 1 contains a correlation plot of GeoQuery aggregated vs. GLocal aggregated values for nighttime lights for the year 2020. The values involving the GeoQuery aggregated values are for USA only, whereas the other values are for all countries. Most of the variables are highly correlated, except for *dmsp*_*ext*, which contains an extension of the DMSP data. This is expected, as the DMSP data is known to have top-coding issues in urban areas, and are also much less precise as compared to VIIRS.

## Usage Notes

While the GLocal datasets provide a clean collection of information to enable development research and policy analyses that rely on international scope and subnational precision, we advise users to exercise judgement in the use of this source and in the interpretation of analyses based on it. A first consideration has to do with the nature of administrative boundaries. As discussed above, while we focus on administrative areas with political and policy meaning in their specific countries, similar efforts have focused on arbitrary “grids” that are not a function of political, economic and cultural features of interest. A separate concern is that local economic and political dynamics of interest may aggregate spatially in ways that transcend both grids and administrative boundaries. Indeed, the UN Statistical Commission has endorsed the “Degree of Urbanization” approach to identify cities, towns, semi-dense areas and rural areas in an internationally comparable manner, leveraging geocomputation methods to outline contiguous areas with meeting population density criteria^47,48^. Finally, users should be mindful that the degrees of political authority in administrative areas coded as being in the same “level” under the GADM classification can be drastically different across national boundaries. While all these considerations invite users to exercise judgement in deciding which levels of analysis to focus on, we expect to add grid-based and urbanization-based spatial aggregations to the GLocal Datasets in the near future.

Another relevant consideration for users to exercise judgement on is whether the information provided in the GLocal dataset is the right measure for a given phenomena of interest. For example, we provide information on temperatures and precipitation from different sources. While this information is important in itself^36^, highlight the importance of focusing on “anomalies” to adequately approximate for weather shocks. Given that estimating such anomalies (and many other transformations of interest) at the administrative level does not require for additional geospatial computations, we opted to only provide zonal aggregations in the GLocal dataset and allow users to exercise produce the transformations pertinent to their analyses.

Similarly, users must be mindful of the type and quality of the variation that is captured by each of the sources in different periods. For example, information on Nighttime lights has been used to approximate for local levels of economic development. However, earlier measures of local nighttime lights based on the Defense Meteorological Satellite Program (DMSP) had problems identifying low emissions in rural areas and also suffered from top-coding problems in relatively developed areas. While data from DMSP was originally released from 1992 to 2013, the program has continued collecting measurements. More precise measures of nighttime lights now come from the Visible Infrared Imaging Radiometer Suite (VIIRS) instrument aboard the joint NASA/NOAA Suomi National Polar-orbiting Partnership (Suomi NPP). There have been efforts to match the DMSP and the VIIRS data to build longer time series at the DMSP unit scale. In the GLocal Dataset, we provide the old DMSP data, the new DMSP data, the VIIRS data and the transformed VIIRS data into the DMSP scale as separate features, allowing the users to consider the specific advantages and drawbacks of the different sources thoughtfully before performing their analyses.

Lastly, users should be mindful that sources that do not originate in satellite observations may suffer from measurement errors that correlate with baseline levels of economic development. For example, it is possible that road density is underestimated in developing areas if existing roads in such environments are less likely to be included in OpenStreetMaps. Moreover, travel times estimates are calculated assuming constant movement at road speed limit, which does not consider how average congestion or environmental factors may influence the impedance of traversing different segments of the road network.

**Fig. 2:**
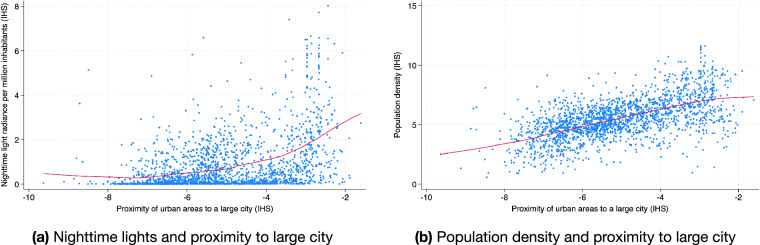
Nighttime lights, proximity to large cities and population density.

Below, we show how cross-sectional information from the GLocal dataset can be used to assess the relationship between the level of development of an administrative area as captured by its measure of nighttime lights radiance (VIIRS) per 1,000,000 people in 2019 and the average proximity (inverse driving time) between its cities and their respective closest city of more than 1,000,000 inhabitants. Panel A of Fig. 2 shows a positive relationship between nighttime lights per capita and proximity to large cities for administrative units at the GID 1. Importantly, Panel B shows that areas close to large cities are relatively dense. While there might be a direct connection between proximity to large cities and living standards, it may be mediated by administrative areas becoming relatively dense. We explore this question by assessing how the linear association between proximity to cities and nighttime lights per person changes after conditioning for local population density. Columns 1 and 2 of Table 2 evaluate this linear association for GID 1 administrative areas after including country (GID 0 Level) fixed effects, showing that the positive relationship between proximity to large cities and nighttime lights is reversed after conditioning for population density. Columns 3 and 4 (5 and 6) evaluate the linear association for GID 2 administrative areas controlling for GID 0 (GID 1) fixed effects, and show that the positive connection between proximity to large cities and nighttime lights per person attenuate by more than 85% after conditioning for population density.

**Table 2:** Relationship between Nighttime lights per person and Proximity to Large Cities.

VARIABLES	(1)	(2)	(3)	(4)	(5)	(6)
	Nighttime light radiance per 1MM people (IHS)					
Urban proximity to large city (IHS)	0.184***	−0.0538***	0.365***	0.0536***	0.273***	0.0322**
	(0.0131)	(0.0137)	(0.00988)	(0.0101)	(0.0140)	(0.0137)
Population Density (IHS)		0.373***		0.512***		0.452***
		(0.0131)		(0.00873)		(0.00992)
Constant	1.742***	−1.518***	4.537***	−0.332***	4.138***	−0.0112
	(0.0683)	(0.128)	(0.0474)	(0.0927)	(0.0652)	(0.108)
Observations	1,975	1,975	10,846	10,846	10,395	10,395
R-squared	0.669	0.770	0.729	0.795	0.845	0.874
Analysis Level	GID1	GID1	GID2	GID2	GID2	GID2
Fixed Effects	GID0	GID0	GID0	GID0	GID1	GID1

Robust standard errors in parentheses.

***p < 0.01, **p < 0.05, *p < 0.1.

To further aid in the comprehension and practical application of the dataset, we have developed a website, glocal.streamlit.app, which offers a user-friendly interface for exploring and visualizing the dataset. Additionally, we have prepared a Jupyter notebook that walks users through an additional example application of the dataset. The code used for these case studies have been stored in this GitHub repository.

## Data Availability

All the relevant code for replicating the different variables in the GLocal datasets are publicly available to users at this GitHub repository. We have added README files that explain how to replicate each component of the study. Note that acquiring each underlying input dataset for aggregation involves different processes. Wherever possible, download scripts have been included to simplify the process of downloading each dataset. However, in some cases, the data is not publicly available, and we have provided instructions on how to request access to the data (mentioned in the codebook).
